# Bile Burns

**Published:** 1902-05-10

**Authors:** 


### BILE BURNS.

Dr. Frederick Griffiths[1] draws attention to the curious fact that vomited bile is sometimes of so acrid a nature as to give rise to "burns" where it comes in contact with the skin of the face. A lady of fair complexion had been taken with an attack of acute gastritis. After vomiting a quantity of food she threw up several ounces of a light yellow and very acrid smelling, watery fluid, and within the course of six hours blebs were raised where this fluid had spattered over her bared shoulder and arm, giving the appearance of a scald. Dr. Griffiths suggests that some of the cases in which injury of the skin of the face has been put down to spilling of anæsthetics may really have been "bile burns." To prevent such results from contact of vomited matters all that is necessary is for the anæsthetist to have at hand a towel saturated with a weak solution of soda with which to wipe the face after the onset of vomiting.

[1] New York Med. Record.

